# Simultaneous Placement of Multiple Airway Stents in Three Patients With Extensive Airway Stenosis: A Case Series

**DOI:** 10.1002/rcr2.70311

**Published:** 2025-08-04

**Authors:** Shogo Toyama, Kohei Fujita, Naoki Fujimoto, Yuta Okada, Saiki Yoshimura, Kiminobu Tanizawa

**Affiliations:** ^1^ Division of Respiratory Medicine, Center for Respiratory Diseases, National Hospital Organization Kyoto Medical Center Kyoto Japan

**Keywords:** airway stenosis, airway stent, flexible bronchoscopy, self‐expanding metallic stents

## Abstract

Airway stenting is a critical therapeutic intervention for managing airway obstruction caused by both malignant and benign conditions. While Dumon Y‐stent placement is sometimes indicated for patients with extensive airway narrowing, facilities capable of performing rigid bronchoscopy are limited. In such cases, the use of multiple self‐expanding metallic stents (SEMS) via flexible bronchoscopy under local anaesthesia presents a valuable alternative. We report three cases in which multiple airway stents were placed simultaneously using this approach. Case 1 involved a patient with stage IV squamous cell lung cancer, in whom the primary tumour caused extrinsic tracheal compression and direct invasion of the right main bronchus. Cases 2 and 3 were diagnosed clinically with tracheomalacia. In all three cases, the placement of multiple SEMS successfully relieved airway stenosis, improved oxygenation, and reduced dyspnoea, without major complications. These cases support the potential of simultaneous multiple SEMS placement as a safe and effective therapeutic option for patients with extensive airway narrowing.

## Graphical abstract

We report three cases in which multiple self‐expanding metallic stents (SEMS) were deployed using flexible bronchoscopy under local anaesthesia. In all cases, stent placement successfully relieved airway stenosis, improved oxygenation, and alleviated dyspnoea without major complications. These cases support the potential role of simultaneous multiple SEMS placement as a safe and effective therapeutic option for patients with extensive airway narrowing.

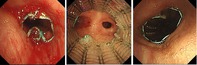

## 1 Introduction

Airway stenting is a crucial therapeutic intervention for managing airway obstruction caused by both malignant and benign conditions [1]. While Dumon Y‐stent placement is sometimes necessary for patients with extensive airway narrowing, facilities capable of performing rigid bronchoscopy are limited. In such settings, the use of multiple self‐expanding metallic stents (SEMS) can serve as a valuable alternative. SEMS can be placed under local anaesthesia, providing a less invasive option compared to surgical intervention and reducing the risks associated with general anaesthesia, particularly in high‐risk patients [2]. There are few published case reports describing the simultaneous placement of multiple airway stents; notable exceptions include cases involving relapsing polychondritis [3, 4] or tracheobronchomalacia [5].

We herein report three cases in which multiple airway stents were placed simultaneously using flexible bronchoscopy under local anaesthesia, as a life‐saving measure.

## 2 Case Series

### 2.1 Case 1

A 68‐year‐old man presented to our hospital with complaints of dyspnoea and cough. He had been diagnosed earlier that year with stage IV squamous cell lung cancer with pleural dissemination. Despite receiving third‐line chemotherapy, the primary tumour had progressively enlarged, resulting in extrinsic compression of the trachea and direct invasion of the right main bronchus. Aside from the cancer diagnosis, the patient had no significant past medical history.

**FIGURE 1:**
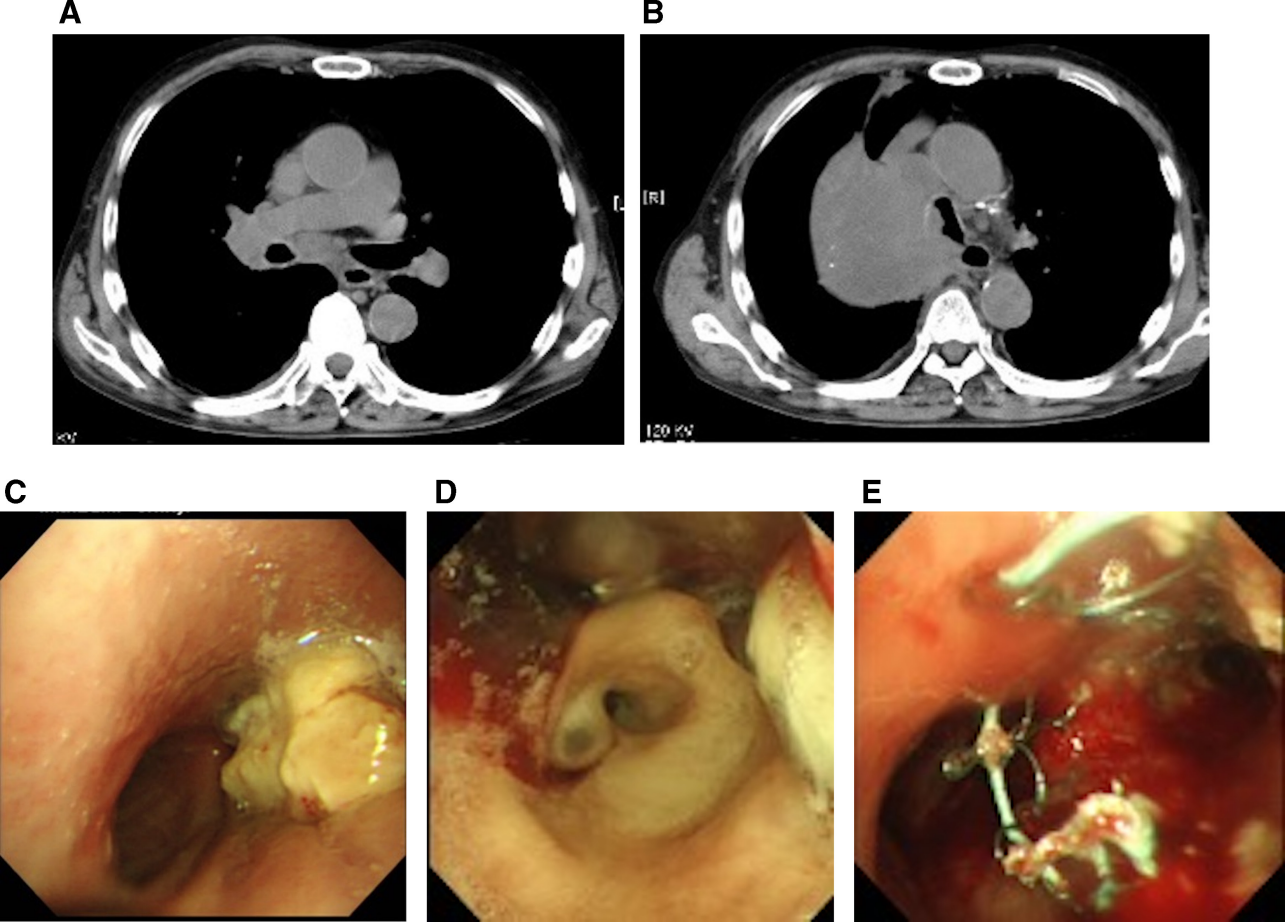
(A, B) Thoracic computed tomography demonstrated extrinsic compression of the trachea and complete obstruction extending from the right main bronchus to the right intermediate bronchus. (C–E) Bronchoscopic findings revealed that the trachea was compressed extrinsically, and the right main bronchus was directly obstructed by the tumour. An uncovered Ultraflex SEMS (14 × 60 mm) was placed in the right main bronchus, and a covered Ultraflex SEMS (14 × 60 mm) was deployed in the trachea.

He was initially hospitalised for obstructive pneumonia and discharged after 2 weeks, but returned 6 days later with worsening respiratory symptoms. On presentation, physical examination revealed markedly diminished breath sounds throughout all zones of the right lung. Peripheral oxygen saturation (SpO_2_) was 86%. Thoracic computed tomography (CT) revealed complete obstruction from the right main bronchus to the right intermediate bronchus (Figure 1A,B). Following administration of prednisolone 7.5 mg, a standby flexible bronchoscopy was performed under conscious sedation with a bite block. Under fluoroscopic guidance, a Bentson wire was advanced into the right main bronchus and stent positioning was marked using skin markers. Over the wire, an uncovered Ultraflex SEMS (Boston Scientific, Natick, MA) (14 × 60 mm) was placed in the right main bronchus, and a covered Ultraflex SEMS (14 × 60 mm) was deployed in the trachea, with its lower end extending to the carina (Figure 1C–E). They effectively relieved the stenotic lesions, improving oxygen saturation. The patient was weaned off supplemental oxygen and discharged on hospital day 13.

### 2.2 Case 2

**FIGURE 2:**
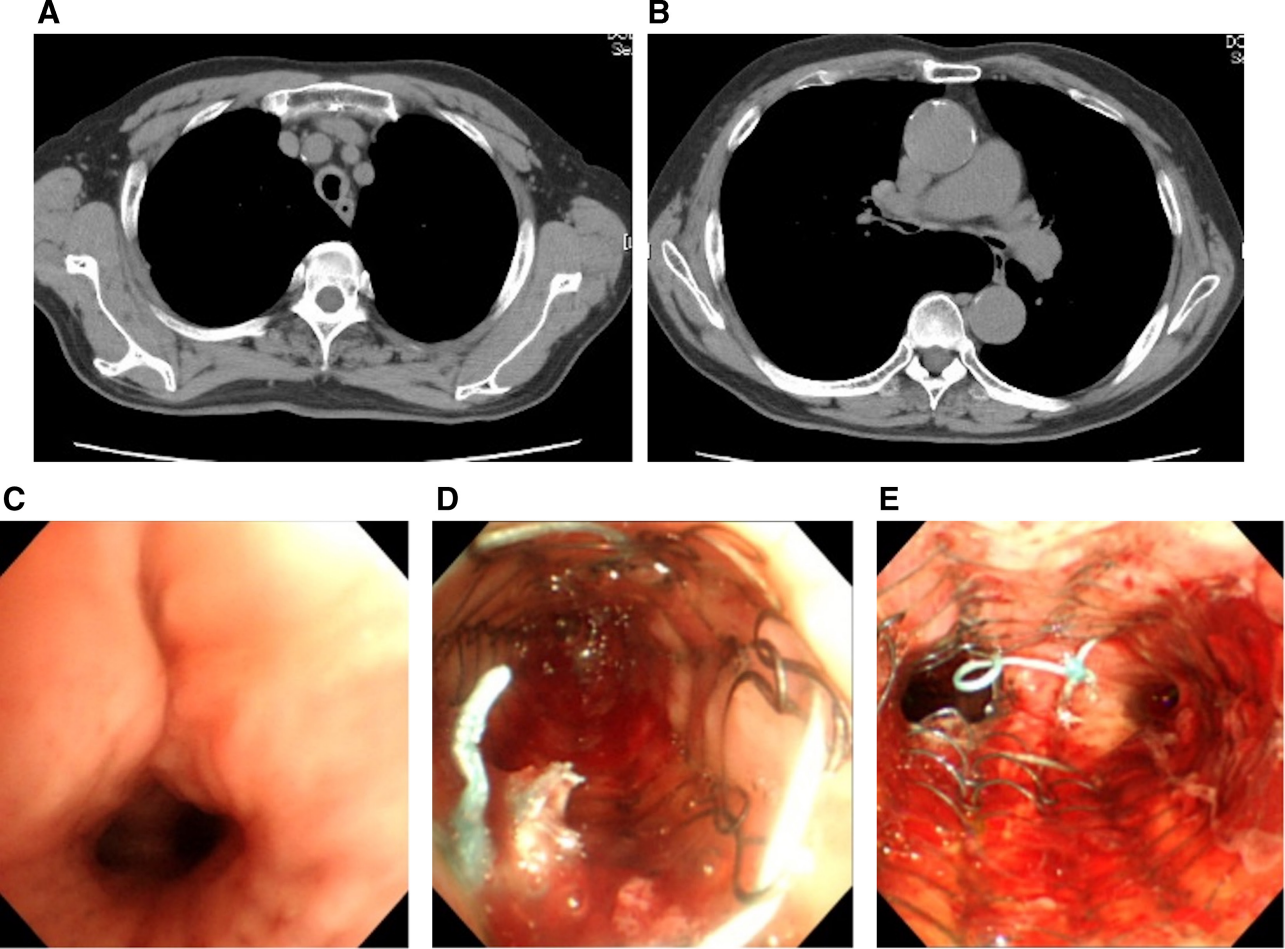
(A, B) Chest CT revealed diffuse bronchial wall thickening. (C–E) An uncovered Ultraflex SEMS (14 × 40 mm) was deployed in the right bronchus intermedius and another uncovered Ultraflex SEMS (20 × 60 mm) was placed in the trachea.

A 76‐year‐old man was referred to our hospital with rapidly progressive respiratory failure. He had a history of chronic obstructive pulmonary disease (COPD) and had been receiving home oxygen therapy at 2 L per minute. The day prior to admission, he had been hospitalised for a femoral neck fracture. Due to worsening hypoxemia that could not be managed with noninvasive oxygen therapy, he was transferred to our hospital, where tracheal intubation and pressure‐controlled mechanical ventilation were initiated. Chest computed tomography (CT) revealed diffuse bronchial wall thickening suggestive of relapsing polychondritis (Figure 2A,B), although bronchoscopic biopsy showed only nonspecific inflammatory cell infiltration. Based on clinical findings, the patient was diagnosed with tracheomalacia, and methylprednisolone 1000 mg was administered for 3 days as steroid pulse therapy. However, the treatment yielded minimal improvement, and ventilatory difficulties persisted. To relieve airway stenosis, flexible bronchoscopy was performed. An uncovered Ultraflex SEMS (14 × 40 mm) was deployed in the bronchus intermedius just below the origin of the right upper lobe bronchus. Another uncovered Ultraflex SEMS (20 × 60 mm) was placed in the trachea, with its lower end extending to the carina (Figure 2C–E). These interventions led to significant improvements in tidal volume and a reduction in episodes of airway obstruction due to sputum retention, although the patient could not be finally weaned from mechanical ventilation.

### 2.3 Case 3

**FIGURE 3:**
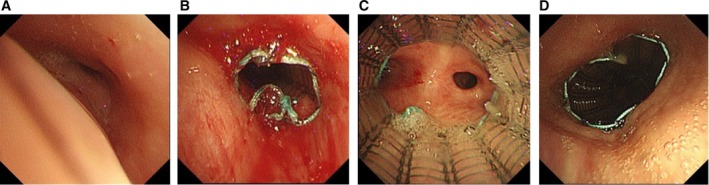
(A) Bronchoscopic findings revealed approximately 90% airway collapse, which was triggered by a mild cough. (B–D) An uncovered Ultraflex SEMS (10 × 30 mm) was deployed in the left lower lobe bronchus, and two uncovered Ultraflex SEMS (20 × 60 mm and 20 × 40 mm) were placed sequentially in the trachea.

A 92‐year‐old man with a history of chemoradiotherapy for oesophageal cancer was admitted for further evaluation of pleural effusion. During hospitalisation, flexible bronchoscopy was performed at the bedside due to a decline in SpO_2_. Nasal high‐flow therapy was administered, as a positive expiratory pressure effect was anticipated. Bronchoscopic examination revealed approximately 90% airway collapse, provoked by a mild cough (Figure 3A). Based on clinical assessment, tracheomalacia was suspected, likely contributing to impaired sputum clearance. To improve airway patency, an uncovered Ultraflex SEMS (10 × 30 mm) was deployed in the left lower lobe bronchus. Additionally, two uncovered Ultraflex SEMS (20 × 60 mm and 20 × 40 mm) were placed sequentially in the trachea (Figure 3B–D). These interventions led to improved oxygen saturation and alleviation of dyspnoea. Several months later, the patient ultimately died of aspiration pneumonia.

In all three cases, the placement of multiple airway stents was completed without major complications such as bleeding, pneumonia, or pneumothorax.

## 3 Discussion

In this article, we reported three cases in which multiple airway stents were placed simultaneously using flexible bronchoscopy under local anaesthesia. All procedures were performed safely and resulted in significant improvements in the patients' respiratory status.

In situations where rigid bronchoscopy is not available, self‐expanding metallic stents (SEMS) placed via flexible bronchoscopy have emerged as a valuable therapeutic alternative. SEMS placement has demonstrated high procedural success rates, with significant symptom relief, improved pulmonary ventilation, and clinical stabilisation of patients [6]. An additional limitation of our study is that self‐expanding metallic Y‐stents, which are compatible with flexible bronchoscopy, were not available at our institution.

In general, we preferred using uncovered stents to preserve ventilation of adjacent lobes and to avoid impairing sputum clearance. However, in cases of direct obstruction by intraluminal lesions, such as tumours—as seen in the right main bronchus of Case 1—a covered stent was selected. In contrast, uncovered stents were used in cases of extramural compression or airway collapse due to tracheal wall weakness.

Notably, our case series included patients with both benign and malignant airway pathologies. Although the Ultraflex SEMS was originally designed for malignant airway obstruction, its application in benign conditions remains a subject of ongoing debate and necessitates further evaluation [7]. However, prior reports have documented its efficacy in treating benign conditions such as bronchomalacia and relapsing polychondritis [3, 4, 5]. The current cases further support the potential utility of SEMS in select benign airway diseases.

According to several single‐centre retrospective studies, approximately 15% of patients undergoing airway stenting required the placement of two or more stents [8, 9, 10]. Regarding efficacy, previous studies have shown that in patients with extensive airway stenosis, additional stenting yielded significant improvements in forced expiratory volume in 1 s (FEV_1_) and peak expiratory flow (PEF) compared to outcomes achieved with initial stenting alone [9, 11]. In terms of safety, no significant difference in survival was found between patients treated with multiple airway stents and those who received stenting at a single site [12, 13]. These findings affirm that multiple stenting can be both effective and safe.

The clinical course and imaging findings of three cases undergoing simultaneous placement of multiple SEMS are presented in the context of current literature. This approach may represent a safe and effective therapeutic option for patients with extensive airway narrowing, including those with both malignant and benign aetiologies.

## Author Contributions

S.T. drafted the manuscript. K.F. and K.T. revised the manuscript accordingly. S.T., K.F., N.F., S.Y., and K.T. managed patients. All authors have reviewed and revised the manuscript for intellectual content. All the authors approved the final version of the manuscript.

## Consent

The authors declare that written informed consent was obtained for the publication of this manuscript and accompanying images using the consent form provided by the Journal.

## Conflicts of Interest

Kohei Fujita is an Editorial Board member of Respirology Case Reports and a co‐author of this article. He was excluded from all editorial decision‐making related to the acceptance of this article for publication. The other authors declare no conflicts of interest.

## Data Availability

The data that support the findings of this study are available on request from the corresponding author. The data are not publicly available due to privacy or ethical restrictions.
